# Are We All Post Traumatic Yet? A Critical Narrative Review of Trauma Among Arab Refugees

**DOI:** 10.1177/27551938251330735

**Published:** 2025-04-13

**Authors:** Osama Tanous, Nadine Hosny, Suad Joseph

**Affiliations:** 1 580389 FXB Center for Health and Human Rights. Harvard University. Boston. MA, USA; 2 Institute de Psychologie, 27213 University of Lausanne, Lausanne, Switzerland; 3 Department of Anthropology, 8789 University of California, Davis, CA, USA

**Keywords:** PTSD, post trauma, Arab, refugees

## Abstract

Post-traumatic stress disorder (PTSD) is one of the most studied, diagnosed, and treated mental health disorders in settings of war and displacement. A large body of literature has questioned the utility of the PTSD framework and its application to traumatic stress among populations experiencing wars, political violence, and displacement that is chronic and on a population level. No review has yet summarized the conceptual alternatives proposed by scholars for refugees in or from the Arab region. Our article reviews conceptual articles from the last three decades that propose alternative frameworks to understand trauma and traumatic stress among Arab refugees in the Arab region. We have identified nine articles that critiqued the applicability of PTSD framework for Arab refugees and/or provided alternative key concepts. Themes such as the individualistic nature of PTSD, the nature and longitude of traumatic stress, the “normalization of traumatic stress,” and the medicalization of trauma have emerged. The articles also discuss social justice as recovery, diagnostic recommendations, and the flow of knowledge production from the Global North to the Global South. Our article expands a growing body of literature critiquing the applicability of Western psychiatric models in settings in the Global South, specifically the Arab region.

Post-traumatic stress disorder (PTSD) is one of the most researched and discussed mental conditions. The Arab region has been for decades a target of political violence, including settler colonial invasions, wars, and proxy wars during the Cold War and “war on terror”.^1,2^ These wars^a^ have produced millions of refugees and internally displaced people that are the subject of extensive studies and interventions focusing on trauma and PTSD.^3,4^ An entire industry of nongovernmental organizations (NGOs), academic researchers, and aid organizations has been created around the trauma and mental health of Arab refugees.^5–9^

While Arab refugees undoubtedly experience traumatic stress, flashbacks, sleep difficulties, memory and concentration problems, or other components of PTSD as defined by the International Classification of Diseases (ICD^10^;) several scholars have doubted the applicability of the PTSD model in the Arab region and its clinical utility in the context of trauma caused by prolonged sociopolitical violence and displacement among Arab refugees.^11–14^

One of the earliest scholars to make this argument that psychopathology, and in this context, trauma, cannot be understood without its context is Franz Fanon, a key precursor for the decolonization scholarly movement.^15^ Originally trained as a psychiatrist, Fanon was largely overlooked in the fields of psychiatry and clinical psychology until recent years. His seminal contributions to the field of decolonization and his call for sociogenic psychiatry,^b,16,17^ however, were confined to the domain of critical theory.^15^ In his work and presented case studies from then colonized Algeria, he described the relationship between people and their surrounding violent environment.^17,18^ He argued that mental suffering in Algeria was inseparable from colonialism, emphasizing how symptoms differed between French settlers and Algerians due to their positionality in the power dynamics of colonialism. His work connects society, health, and power, emphasizing that without recognizing these connections, we cannot truly grasp the causes and expressions of mental suffering.

Fanon was among the first scholars to examine the connection between psychiatry and structures of power and its impact on the conceptualization of psychopathology, including trauma. Fanon's criticism is still relevant since this relationship continues to define how trauma is conceptualized and studied.^15^ He also cautioned against importing Western psychiatric frameworks to different sociopolitical and cultural contexts, arguing that “modern” psychiatry is a product of a very particular historic, economic, and epistemological legacy of Europe and North America.^15^ In adopting such models, scholars and practitioners dismiss cultural and social sources of distress and pathologize normative reactions to violence and oppression.

In Palestine, for example, scholars have noticed that trauma discourse flattens and derails the experiences of social suffering by medicalizing and pathologizing the normative reactions of those living under military occupation instead of identifying the military occupation itself as social pathology.^19–21^

In the Global North, a similar critique has been accompanying the PTSD discourse since its inception. In her recent book, “*Combat Trauma: Imaginaries of War and Citizenship in Post-9/11 America*”,^22^ Nadia Abu El Haj traces the formation and mutation of “post-Vietnam syndrome,” later known as PTSD. Abu El Haj showcases how the concept was created and used in the 1960s and 1970s as a psychodynamic theory of combat trauma and was saturated with a radical critique of American imperial wars. The term *PTSD* was used and advocated for by anti-war psychiatrists and veterans. In 1980, during the aftermath of the Vietnam War, driven by the need to codify and reimburse traumatic disability, a shift in the conceptualization of traumatic stress occurred to focus on the victimhood of veterans. The American Psychiatric Association (APA) added “Post-Traumatic Stress Disorder” to the third edition of its *Diagnostic and Statistical Manual of Mental Disorders* (DSM-III), describing a cluster of symptoms that plague some survivors of traumatic events, including nightmares, flashbacks, depression, dissociation, and hypervigilance.^23^

Consequently, the trauma discourse became increasingly depoliticized, that is, detached from tackling the political origins and root causes of the trauma inflicted.^24^ This shift, and the creation of PTSD as an official psychiatric medical category in 1980, has pathologized the behaviors of anti-war soldiers and veterans and silenced political and ethical discussions on the connection between war and trauma led by anti-war psychiatrists and veterans. By the end of the twentieth century and the beginning of a new era of American “wars on terror,” PTSD among soldiers and veterans had completely transformed into a clinical and psychiatric condition requiring clinical intervention in the form of therapy or psychotropic medications. Aiming only to restore the “functioning” of soldiers, PTSD treatments became part of the cost of war in a way that is separated and eviscerated from its anti-war political and ethical origin.^22^ Abu El Haj argues that this global misuse of the trauma discourse went hand in hand with a global change in left politics that has changed its focus from a fight for justice, equality, and redistribution of wealth to “care for human suffering” (^22^ p. 16), thus also depoliticizing the left politics from tackling upstream causes of injustice to merely treating downstream symptoms. When it comes to refugees, this is further enforced by the concept of “care for those who fall outside the ambit of care by nation states.”(^22^, p. 17)^c^

Derek Summerfield also notes that in Global North societies, PTSD became a popular diagnosis that emphasizes individual victimhood, medicalized pathologies, and claims for compensations.^25^ Years later, in her work in Lebanon, Moghnieh showed that the PTSD discourse was exported to conflict zones in the Arab region, stating that the PTSD is treated by humanitarian programs and aid granting bodies as the only legitimate forms of war-related suffering.^26^ This spread of the PTSD discourse by humanitarian programs to war-affected regions has sanitized and reduced the horrors of war to a mere technical issue of individual mental homeostasis amenable to repair using medical and psychological interventions.^25^

It is important to follow this genealogy of trauma and the use of PTSD in clinical medicine and public health, especially as it spreads globally and becomes imposed on populations that are significantly different from the Western milieus where it was initially developed and used. Despite several revisions and iterations, by large the war trauma's conceptualization in most Euro-American/Global North contexts remains individualistic, single event oriented, short term, outside of the country's borders, and concerning mainly soldiers.^24^ However, for many Palestinians, Iraqi, Yemeni, Syrians, Libyans, Sudanese and other people living in the Arab region, and in the Global South in general, war trauma is experienced as a constant and pervasive reality of daily life with overwhelmingly severe difficulties.

Although several researchers have proposed models aimed at moving beyond the “post” traumatic stress disorder model in various regions of the Global South, so far no critical synthesis has been conducted to specifically address models of trauma in the Arab region.^27,28^ Our observation of the inconsistencies of the trauma discourse and the way it was imposed on scholars and clinicians in the Arab region is the motivation behind our scholarly work in the Transforming Refugee Mental Health (TRMH) working group.

## Aims and Methods

This article is a narrative conceptual review, with the aim of reviewing literature of the past three decades addressing the framing or conceptualization of traumatic stress in Arab populations. The Arab region was chosen as the site of examining knowledge production on refugee trauma because it is one of the regions with the highest rates of ongoing political violence and upheaval that produces more refugees and internally displaced people than other regions of the world.

Our article is part of our work in the TRMH working group,^29^ part of the University of California (UC) Davis Arab region Consortium (UCDAR), an interdisciplinary collaboration between UC Davis and five universities in the Arab region aiming to pioneer scholarship in the social sciences. Our program includes a multidisciplinary team coming from disciplines such as cultural and medical anthropology, social and war psychiatry, clinical psychology, and public health. It aims to explore and redefine refugee mental health in the Arab region, in vulnerable populations displaced by wars and political violence.^8^

Our work aims to map and review critical knowledge production of empirical and conceptual studies using alternative trauma models or challenging the dominant and reductionist Western conceptualization of trauma and the reflexive use of PTSD in the Arab region. Our first review that examines empirical studies was published elsewhere.^30^ Please see the Supplementary Material for the search strategy and selection process.

In this narrative review, we include the very few conceptual studies that engage in critiques of Western models of trauma in the Arab region and aim to provide alternative perspectives.^30^ The first two authors employed thematic analysis to identify recurring themes within the nine conceptual papers. Articles were divided between both authors, and they both held regular meetings to discuss, reach consensus, and form themes and findings.

## Findings

After a thorough search and review, only nine eligible conceptual research articles were found to challenge Western conceptualization of Arab refugee trauma in the Arab region (see Table 1). In the following sections, we present and critically discuss the main themes that emerged from our thematic analysis and were consistent over the nine articles included in the analysis. When reporting the subject of trauma in our findings, we try to use the word *person*, not *individual*. In our previous review,^30^ we discussed that the conceptualization of the word *individual* is embedded in Western cultural, economic, political, and legal history. The word, while bound to a specific context, has universalized itself, ignoring other modes of selfhood where the autonomous and bounded self is the norm^31,32^ We only use the term *individual* in articles that we have included.^d^

**Table 1.** Conceptual Articles Challenging the use of PTSD among Arab Refugees.

1	Giacaman, Rita. “Social suffering, the Painful Wounds Inside.” *American Journal of Public Health* 107.3 (2017): 357–357.
2	Giacaman, Rita. “Reframing Public Health in Wartime: From the Biomedical Model to the ‘Wounds Inside.’” *Journal of Palestine Studies* 47.2 (2018): 9–27.
3	Giacaman, Rita, Yoke Rabaia, Viet Nguyen-Gillham, Rajaie Batniji, Raija-Leena Punamäki, and Derek Summerfield. “Mental Health, Social Distress and Political Oppression: The Case of the Occupied Palestinian Territory.” *Global public health* 6, no. 5 (2011): 547–559.
4	Yudkin, Joshua S., Parul Bakshi, Kelsey Craker, and Sari Taha. “The Comprehensive Communal Trauma Intervention Model (CCTIM), an Innovative Transdisciplinary Population-Level Model for Treating Trauma-Induced Illness and Mental Health In Global Vulnerable Communities: Palestine, a Case Study.” *Community Mental Health Journal* (2022): 1–11.
5	Aarethun, Vilde, Gro Mjeldheim Sandal, Eugene Guribye, Valeria Markova, and Hege Høivik Bye. “Explanatory Models and Help-Seeking for Symptoms of PTSD and Depression Among Syrian Refugees.” *Social Science & Medicine* 277 (2021): 113889.
6	Afana, Abdelhamid “Problems in Applying Diagnostic Concepts of PTSD and Trauma in the Middle East.” *Arab Journal of Psychiatry* 23 (2012): 28–34.
7	Amawi, Noor, Richard F. Mollica, James Lavelle, Ossama Osman, and Laeth Nasir. “Overview of Research on the Mental Health Impact of Violence in the Middle East in Light of the Arab Spring.” *The Journal of Nervous and Mental Disease* 202, no. 9 (2014): 625–629.
8	Kira, Ibrahim A. “Etiology and Treatment of Post-Cumulative Traumatic Stress Disorders in Different Cultures.” *Traumatology* 16.4 (2010): 128.
9	Wells, Ruth, Catalina Lawsin, Caroline Hunt, Omar Said Youssef, Fayzeh Abujado, and Zachary Steel. “An Ecological Model of Adaptation to Displacement: Individual, Cultural and Community Factors Affecting Psychosocial Adjustment Among Syrian Refugees in Jordan.” *Global Mental Health* 5 (2018): e42.

### The Impact of Trauma Surpasses “Individual” Distress

Most papers saw that war and political violence are pervasive and extend to virtually everyone in the Arab region. The war reaches the homes and communal spaces of civilians and is at their doorsteps.^20^ As Giacaman and colleagues argued, trauma was often framed as a collective or communal, rather than an individual experience, that results from brutal and large-scale exposure to war and violence causing suffering and humiliation on a national level. The exposure, including but not limited to bombing, shooting, killing, injuring, shelling of neighborhoods, tear gassing and sound bombs thrown indiscriminately, especially around checkpoints, were shared, not individual, isolated, experiences.^19,33^ The articles noted that trauma vulnerability and resilience operate not only on individual but also on communal levels.^34^

Afana^35^ argued that PTSD research has not focused enough on how traumatic events are experienced by families, subgroups, communities, or cultures in the Middle East, and how the shared response of these groups affects and is affected by the individual experience. As collective trauma is influenced by social processes such as meaning making, its complex determinants should be considered when formulating clinical diagnoses and interventions. Kira's model of cumulative trauma makes the point that in contexts of collective trauma, traumatic events are often experienced and expressed in relation to one's social identity and role within the community, leading to symptoms that are more focused on social and interpersonal aspects.^36^

Wells and colleagues,^37^ on the other hand, adopt a functional approach and propose an ecological/transactional model that recognizes how traumatic exposure to war and displacement severely disturb social systems and thus undermine the overall stability and coherence of a society. We note here that this approach has often been critiqued for its tendency to reduce psychological processes, such as adaptation, to functional roles, while overlooking salient factors that influence intrapsychic processes, such as constantly changing societal norms and inequalities.^38^ However, the model proposed by Wells and colleagues also attempts to go beyond functionalist assumption of social stability. They explain that adaptive processes and individual resource building are mediated by “identity markers,” which include gender, age, ethnicity, sexuality, disability, trauma history, and financial and sociopolitical status. While doing so they acknowledge that such factors have an ever-changing and dynamic nature, as they are based on the interaction between individuals and their social context in war and displacement settings.^39^ This model can explain how communities respond to challenges such as human rights violations, and in turn shape their collective response.

Wells and colleagues base their model on transactional models, which describe how people use available resources to cope with adversity and develop a niche for adaptation and cultural inheritance in which the individual and the collective are intertwined. Their model includes four nested layers: intra-individual, family/peers, society, and culture.^37^ To mirror this concept of layers, Afana studied^16^ Palestinian idioms of distress like “Sadma” صدمة (trauma as a sudden blow with immediate impact), “faji’ah” فاجعة (tragedy), and “musiba” مصيبة (calamity) as culture-specific reactions to trauma^35^ that impact not only one's family functioning but disrupt the overall functioning in all four layers, thus creating a social crisis.

### The Nature, Location and Longitude of Trauma

The nature and scale of the traumatic events was also a recurrent theme in each article. While the PTSD model emphasizes a single traumatic event in the past, the articles emphasized that the nature of trauma in the Arab region is ongoing. There is an ongoing exposure to a variety of forms of violence, people live in “chronic warlike conditions”^33^ that lead to a loss of protection embedded in the ways of life. Continuous exposure to war profoundly affects physical, mental, and social health by producing a relentless anxiety, exhausting hypervigilance, loss of control, helplessness, and, ultimately, a chronic social crisis.^34^ Entire communities experience severe insecurities, instability, uncertainties, deprivation, loss of dignity, and humiliation that lead to deep internal wounds of survival. The events were seen as causing social suffering on the continuum of ease–disease that is distinctively different from yes/no diagnostic cutoffs of PTSD. As Giacaman argues, the exposure is cumulative, and the survivors of wars oscillate back and forth on the spectrum of ease–disease.^20,33^

Another distinct difference about the traumatic event is that while in the PTSD model the triggering event is “exceptional,” for refugees and people displaced by wars, the ongoing wars become everyday experiences. It becomes the ordinary rhythm of life that reaches civilian homes; the “home front becomes the battlefront”^33^ to create sadness and misery.^40^ People do not only experience the violence of war as a traumatic event, but also the destruction of the entire cultural, material, and social worlds they live in that define values and roles. In that sense, war creates trauma and destroys the ability to deal with it by destroying the coping or protective mechanisms people and communities have. The social suffering and injustices that survivors of war trauma report are not scattered personal events but rather connected to political points in history such as the “Nakba”^41,42^ النكبة (catastrophe) and different wars for Palestinians, the War in Iraq and Syria, and other political events. Other scholars have also made this distinction between the experience of living in violence versus the experiencing of encountering it. These distinct experiences predicate different forms of psychological and social suffering.^43^

The changes after the traumatic events (refugeehood, displacement) also continue for decades, in itself creating and worsening ongoing trauma due to precarious life in refugee camps or in a state of refugeehood and dependency on international aid.^19,40^ For example, Arethun and colleagues found that Syrian refugees attributed their symptoms to various cumulative stressors, including trauma, family, and financial problems. Some mentioned overcrowded or unsafe housing, poverty, separation from family members, and caring for dependents in difficult circumstances. Stressors that were found to persist or increase over time included loss of social networks, culture-related difficulties, and bureaucracy-related problems.^44^ Afana argued that such ongoing context stressors should be incorporated in trauma models, as empirical studies show that being worried about personal safety and the ability to fulfill basic needs had a more significant impact on the severity of PTSD symptoms than the severity of the traumatic or violent events themselves.^40^

In his 2010 work, Kira also suggested that the traditional PTSD model assumes a one-time traumatic event and focuses on symptoms that are immediately observable and measurable. However, in situations of ongoing and repeated trauma exposure, such as in many war-affected areas, the symptoms of PTSD may become cumulative. Thus, the traditional PTSD model may not fully capture the complexity of experiences and symptoms that people may have.^13^ Kira proposes the post-cumulative traumatic stress disorders (PCTSD), which is the result of multiple or ongoing traumas, or other vulnerability contexts—for example, social and economic marginalization or exclusion in which people are exposed to multiple types of interpersonal trauma, as opposed to a single, personal traumatic event.

### The Normalization of Traumatic Stress

The articles show that participants are aware of their own distress, as Aarethun and colleagues point out that participants recognized and identified with symptoms of PTSD, but never explicitly named it: “Participants also normalized symptoms, however normalizing symptoms of PTSD does not necessarily entail a de-emphasis on the character's psychological pain” (^44^ p. 4). By *normalization*, the authors meant that in contexts of protracted violence, anxiety, depression, and other PTSD symptoms are seen as enduring states that people adapt to and learn to live in.^40^ Multiple causes were given to these processes of normalization, like the prevalence and enduring nature of violence and other stressors, or the communal nature of exposure.^37,40,44^ Another reason given for normalizing symptoms, for example, is that Palestinians normalize their suffering within the political context of their experiences. While the distress remains, this normalization allows for positive actions to be taken to alleviate their distress and change living conditions instead of seeking psychological aid.^40^ Articles propose that a fine balance needs to be struck between maintaining and incorporating the wider sociopolitical context of distress/symptoms conceptualization and not dismissing personal experience of suffering and the burden of these symptoms on people.

We use the term *normalization* here in reference to its use in the articles presented to describe the effect of prolonged exposure to violence and the process of habituation, how it changes notions of normalcy, and the conceptual and clinical implications of this process. We are, however, aware of the critique of the concept of “normalization” and the history of its use as a tool of power in determining norms and deviance, as well as in deciding which narratives are included or marginalized.^45,46^

### The Medicalization and Decontextualization of Trauma

Studies emphasized the ill fitness of biomedical individualized instruments to capture shared experiences of trauma.^33^ Two articles^19^^,^^33^ raised doubts about the capacity of disciplines like clinical medicine and psychology—which typically concentrate on individual cases in a clinical context—alone to adequately comprehend and conceptualize shared experiences in their models of trauma. They suggested that disciplines such as public health, which emphasizes the population level, will be able to offer insights for understanding and recovery of shared trauma,^19^ as these fields take into account the “causes of causes”.^20^ Researchers argue that at this point, it has become obvious to practitioners and scholars that individualized treatment with counseling and medications could not address the “underlying causes of ongoing collective trauma” (^33,^ p. 15) after witnessing the limited efficacy of repetitive short-term cycles of aid projects lasting two to three years.^19^

And thus, studies propose that medicalizing the entire process—from conceptualizing war trauma as a biomedical illness to medicalizing recovery with multiple treatments—framing war suffering as a mental condition rather than a social and political issue became “questionable, if not downright harmful”(^33,^ p. 16).^40^ Some authors showed concern as terms like *PTSD*, *psychosocial*, and *counselling* rapidly became hegemonic points of reference in this “new realm of humanitarian concern”(^19^, p. 549). The authors were also concerned with the fact that even though many communities have relied on family support and community aid, international agencies adopted Western-designed trauma programs that were seen by local practitioners as out of context.^19,34,40^

The articles recommended a change of perspective from one that is “outcome based on medical indicators, injury and illness”(^19^, p. 556) to a framework based on social suffering and human rights violations experienced by ordinary people. This move should also include aid agencies that should shift the short-term humanitarian response to a long-term development with local partners combined with international advocacy for the respect of human rights and the restoration of justice.^19^ Some of the articles suggested specific communal trauma intervention models that focus on shared trauma^34,36^

### Social Justice as Recovery

In continuation with the previous section, and with the recommendation to depart from biomedical models, the authors emphasized the importance of the context. Recovery was seen by the authors not as an individual process but as an etiological reconstruction of a disturbed social world and injustice causing subsequent suffering. In such a paradigm shift, genuine recovery would correlate with social justice, access to resources, strength, cultural stability, and social support, among other factors.^20,36^ The articles called for a shift from international aid policies based on medical mental health determinants and outcomes to policies that combine a public health approach to mental health with international advocacy for human rights and justice.^19^ The articles emphasized that it is important to differentiate between mental disorders and the social suffering of war: “Attempts to measure the social suffering of populations affected by complex political emergencies are therefore part of an overall approach that places the demands for rights and justice at the center of global health” (^19^, p. 555). The aim of the intervention should be “the reversal of historical injustice” (p. 555). This is a priority for the protection of mental health in war affected regions.^19^

Moreover, in reconstructing a social world, some authors suggested that part of recovery was the creation of routine and a sense of normality that defies chaos and hopelessness: “Social capital, in the form of a tight network of family support, peers, friends, caring adults, clubs and schools, nurtures and sustains the sense of optimism and hope” (^19^ p. 556).

### Diagnostic Recommendations

Addressing and reversing the mental health effects of war-related trauma requires tackling root causes of armed conflict, colonial legacies, and settler colonialism in order for communities to flourish in a safe, stable, and healthy environment.^e^ However, the authors were not disillusioned about the fact that in a region marked by expanding warfare and displacement, some of these recommendations become impractical in the short term, and pragmatic and actionable steps are needed. The articles offered several concrete recommendations for scholars working on traumatic stress among Arab refugees. They suggested and, in some cases, developed new measurements for health-related quality of life that are specific to the local context, such as a measurement for humiliation, human insecurity scale, scale of distress, and scale of deprivation. Those scales capture more accurately the lived context of the studied communities than the World Health Organization's (WHO) standardized quality of life scale, which was seen as weak on the social domain and devoid of a political domain.^33^

Local idioms of distress assist scholars in the understanding of shared suffering as complementary to but distinct from individual suffering. Such idioms are not diagnostic entities that require treatments but terms through which distress is expressed and social support is sought and mobilized.^19,37,40^ Afana^40^ emphasized that the same PTSD symptom endorsed by two individuals from different cultural backgrounds does not necessarily have the same meaning. Moreover, within the same culture, the same PTSD symptom will have various representations depending on the severity of impact on communal health, as we have seen in the case of *sadma* (trauma as a sudden blow with immediate impact), *faji’ah* (tragedy), and *musiba* (calamity).^40^ The use of idioms and metaphors to describe psychiatric symptoms reflects a socially based vocabulary. From the perspective of clinical phenomenology of symptoms, certain authors added that PTSD or responses to exposure to violence among Arab populations is a collection of symptoms (including PTSD, anxiety, depressive, somatic, and dissociative), and research should focus on the heterogeneity of responses to trauma to develop more appropriate conceptual models and treatments that demonstrate cultural competence and cultural humility.^36,37,40,47^

### Knowledge Production

For the authors, it was evident that a lack of local high quality research on the topic exists, as “When a nation is struggling with day-to-day survival, the development of evidence-based mental health policies and services is unlikely to be a priority” (^19^ p. 357). It was clear to the authors that the flow of information and research in the forms of diagnostic tools and intervention in global mental health is unidirectional, from high income countries of the Global North to passive and silent recipients in the Global South. As Yudkin and colleagues argue, “In fact, post-traumatic stress disorder (PTSD) was originally conceived and defined within the population of U.S. war veterans, yet it is regularly applied to heterogeneous populations and experiences across the globe” (^34^, p. 300) This leads to a Western hegemony in coining and distributing scientific concepts and reproducing coloniality, the epistemic effects of colonialism. Such coloniality that imposes Western sociocultural-moral constructs onto “theoretically marginalized” communities was seen as unethical and carrying dangerous adverse outcomes.^34^

In line with this observation, the articles we reviewed also highlighted that to better understand the impact of violence on human suffering in the diverse societies of the Arab region, local researchers should prioritize cultural validity and local community interests in their qualitative and quantitative studies. In this process of “decolonization” and emancipation, Arab researchers should also use scientific methods that reflect the region's values, not replicate studies designed for different cultural contexts in the Global North. Throughout the articles, the authors emphasized the importance of having Arab researchers develop their own research methods and priorities, taking into consideration the cultural and sociohistorical values of their patients and communities. Conducting studies that could translate into health benefits within their societies is crucial, and will also contribute significantly to the global but multipolar trauma field.^40,48,49^

## Conclusion

Our review has identified a nascent yet evolving body of literature rejecting and critically engaging with the hegemonic Western conceptualization of traumatic stress among Arab refugees. It is evident from the discussed studies, and from a large body of literature, that PTSD is not a “disease” in the pure biomedical sense with universal diagnostic and therapeutic tools. One cannot diagnose and treat PTSD in the same manner as diabetes mellitus or hypertension using the similarly medicalized and quantified metrics, screening tools, and treatments. Scholars have highlighted how applying the PTSD framework uncritically can be unbeneficial and even harmful, as it helps to pathologize the reactions of ordinary people to abnormal events instead of exploring the contours of psychological damage to populations experiencing abnormal living conditions and traumatic experiences on a population level. The PTSD framework flattens the experiences of refugees who have traumatic stress, and it limits the organic and local intellectual and academic engagement with the mental consequences of the political violence that tears apart the fabric of societies and produces displacement, refugeehood, poverty, and unlivable places.

The inapplicability of the “post” in PTSD is becoming more and more established from scholarship on traumatic stress following political violence in the Global South. While most American and European soldiers who encounter war trauma go back to the safety and security of their homelands to process and be treated for PTSD, Arab refugees exposed to war trauma continue to experience the traumatic stress and the devastation of war that has destroyed the social, economic, and political structures of societies. A serious and honest engagement with the traumatic stress of Arab refugees, or for that matter, of most refugees in the Global South, must take into consideration the political aspects of the wars that have ravaged the region and the continued traumatic stress of worrying for oneself and one's family and society—all while entire nation states are collapsing and failing to provide the minimally decent needs for human life.^50^ These insecurities, completely absent in the PTSD framework, ought to be integrated in any serious analysis and engagement with refugee mental health. Our aim is not to completely de-medicalize traumatic stress. We do not argue that patients with traumatic stress should not receive medical and psychosocial support; instead, we contend that a fine balance needs to be struck between under- and overmedicalization of trauma-related distress. What we do suggest is that research and praxis on traumatic stress should investigate its root causes and integrate holistic frameworks.

The extensive body of literature in public health that studies racial, class, or gender health disparities by addressing the social and political context in which those identities were constructed and maintained and how they drive health inequities^42,51–57^ provides an example of how mental health among refugees can be studied to incorporate the root causes that have produced uprootedness, refugeehood, impoverishment and sustain traumatic stress.

The scarcity of the papers that we found show that the Arab region is not a site of knowledge production that challenges the Western conception of PTSD among refugees. We believe that an essential obstacle to the expansion of the field of critical refugee trauma studies in the Arab region is the coloniality dominating the social and health sciences.^58^ As Giacaman suggests in a recent article, despite calls for decolonization, the architecture of knowledge production continues to be dominated by English-speaking neoliberal institutions that commodify research and teaching and control through funding what should be researched and how.^59^ Subsequently, Arab universities reproduce Western coloniality and center periphery relation by adapting promotion and tenure programs for scholars based on publications in English and in Westernized forms of knowledge production.^60^ This landscape limits critical and organic engagement with a topic as complex and multidisciplinary as traumatic stress following political violence.

Several scholars in the Arab region are exploring the effects of the communal protracted violence in the region and the necessity to move beyond the trauma/resilience binary (e.g., Lamia Moghnieh's work in Lebanon and Rita Giacaman's work in Palestine^26,43,61,62^;). However, we conclude clinical trauma researchers may still be left with lingering questions such as: In such contexts, how do we conceptualize and measure psychopathology? What is considered normal and what is considered abnormal? How can we prevent the occurrence of over- or underdiagnosis in Arab refugee contexts? In what manner may we intervene?

We provide a synthesis of the existing literature and, whenever possible, answers to such questions. Empirical evidence is still needed to provide a comprehensive response to these queries. However, based on our review, as a multidisciplinary research team, we can offer the following potential directions or inquiries to follow.

Based on concepts such as the continuum of ease and disease^33^ and the current direction in global mental health, which measure burden of mental distress based on functioning,^63^ we can build local models that understand the spectrum of well-being and illness and prioritize a transdiagnostic approach based on functioning. Should we abandon the PTSD model by adopting a symptom-led approach, and potentially develop distress checklists that encompass pertinent categories and idioms of distress, as done in other war-torn regions (e.g., Afghan Symptom Checklist;^64^ South Sudan Mental Health Assessment Scale^65^)?

Another potential option is to prioritize the development of alternative models through bottom-up research and anthropological fieldwork. This can be achieved by building upon existing models such as Kira's Cumulative trauma model^66^ or Straker's model of continuous traumatic stress,^28,67^ developed and utilized with other populations involving prolonged structural violence. The aim is to create clinical models that are culturally and structurally relevant, which include a bandwidth of trauma-related symptoms and yet conceptualize them differently. These models can then be used in clinical and diagnostic settings.

While this paper was being finalized, the Gaza Strip was being subjected to some of the deadliest and most intensified warfare in modern history, what many experts have affirmed is a genocide.^68^ Up until the time of writing, the Israeli Army killed more than 50,000 Palestinians, thousands of children became orphans, and most of the housing, educational and health care facilities in the strip were ravished, creating further mass displacement on an already displaced refugee population.^68–70^ The survivors of the genocide in Gaza will have to live with the unbearable suffering of witnessing their children, parents, or family members burnt, starved, killed under the rubble, or dismembered.^71–73^ Our article emphasizes that approaching this monstrous scale of traumatic stress in research and practice cannot be divorced from the political reality of the colonization of Palestine. Testing for PTSD symptoms in more than two million Gazans, survivors of genocide, cannot provide us any meaningful insight into the state of traumatic stress in such dystopic conditions. Hence, scholars and practitioners should develop conceptual models that accommodate the effects of such traumatic stress while taking an active role in situation justice and decolonization within their work, and address the root causes producing this misery and advocate for ending it.

## Supplemental Material

sj-docx-1-joh-10.1177_27551938251330735 - Supplemental material for Are We All Post Traumatic Yet? A Critical Narrative Review of Trauma Among Arab Refugees Supplemental material, sj-docx-1-joh-10.1177_27551938251330735 for Are We All Post Traumatic Yet? A Critical Narrative Review of Trauma Among Arab Refugees by Osama Tanous, Nadine Hosny and Suad Joseph in International Journal of Social Determinants of Health and Health Services

## Notes

a. By wars in the Arab regions, we do not only mean the warfare itself but the entire destruction of societies, economies, infrastructures, and resulting impoverishment and brain drain in the region where political violence and instability has been a hallmark for decades.

b. Sociogeny and sociogenic psychiatry emphasize the impact of social and cultural factors on mental health and illness. The approach considers social structures, relationships, and cultural norms in addition to biological and psychological factors when studying mental disorders and their treatment.

c. With the global rise of fascism and right wing ethnonationalism, we are seeing an abandonment even of politics of “care for human suffering” as more populations, especially Arabs, Muslims, and migrants, are seen either as enemies of the West and thus have brought their suffering upon themselves or simply disposable and unworthy of care. Acts such as the defunding of United Nation Relief and Works Agency For Palestinian Refugees in the Near East and the ambivalence towards the massacres and starvation in Gaza and Sudan are emblematic.

d. Please refer to Hosny and colleagues^30^ for a more detailed critique of the term *individual*.

e. This logic mirrors what we saw in the previous century with infectious diseases, where preventive measures and addressing root causes aiming to create a healthier and more hygienic environment through vaccines, sanitation, hygiene, and water and sewage infrastructure reduced the burden of infectious diseases far more than antibiotics and treatments.
