# Supplementary material for: Are We All Post Traumatic Yet? A Critical Narrative Review of Trauma Among Arab Refugees
Source: Int J Soc Determinants Health Health Serv. 2025 Apr 13;55(3):341–51. doi: 10.1177/27551938251330735 (PMC12171028; doi:10.1177/27551938251330735)
Supplement: sj-docx-1-joh-10.1177_27551938251330735 - Supplemental material for Are We All Post Traumatic Yet? A Critical Narrative Review of Trauma Among Arab Refugees [file sj-docx-1-joh-10.1177_27551938251330735.docx]

Supplementary material

Arksey and O’Malley Framework for Scoping reviews

**Stage 1:** Identify the Review Question

We ﬁrst conceptualized our research question based on a broad but rapid literature review in three successive “blast searches,” which revealed that studies of Arab refugee trauma are carried out across a wide variety of disciplines and professions. This insight prompted us to choose a transdisciplinary, interprofessional, team-based approach.

Our five team members encompass diverse roles, expertise, skills, and professional contexts, which included a cultural anthropologist, a medical doctor trained in public health, a clinical psychologist, a psychiatric epidemiologist, and a senior librarian. The entire team trained in scoping and systematic reviews, and for six months had bi-weekly meetings conceptualizing our review question and navigating across different disciplines.

We defined refugees as Arab males and females aged 21 years or older, displaced within the Arab region, regardless of their official immigration status.

**Stage 2:** Identify Relevant Literature

We searched the following databases: Scopus, MEDLINE, PubMed, PsycInfo, PTSDPubs, Web of Science, Anthropology Plus, and Global Health. For Arabic literature, the following databases were searched: Al-Manhal, Al Mandumah, Arab Citation Index. Searches were conducted for peer-reviewed studies published between 1990 and 2023 and written in English and/or Arabic.

The construction of the search strategy was led by a senior librarian at University California Davis Between December 2021 and May 2022; we performed three exploratory “blast searches” along with backward and forward citation reviews to probe search terms and refine the research question and strategy. The following search terms were chosen:

**Table 1**

Search Terms

| **Search terms** |
| --- |
| **Search concept 1: Arab Countries**  Arab / Arab speaking / Algeria / Bahrain / Djibouti / Egypt / Iraq / Jordan / Kuwait / Lebanon / Libya / Morocco / Oman / Palestine / Qatar / Saudi Arabia / Somalia / Sudan / Syria / Tunisia / United Arab Emirates / Yemen |
| **Search concept 2: Status or Exposure**  Refugee / post-conflict / conflict affected / asylum seeker / internally displaced person violence / war |
| **Search concept 3: Distress**  trauma / traumatic / post trauma / PTSD / psychological distress / psychological symptom / psychological dysfunction / emotional distress / psychiatric symptom / psychiatric condition/ / idiom of distress / cultural concept of distress / explanatory model for mental health and illness / explanatory model for trauma / assessment / evaluation / diagnosis |
| **Search concepts 4: Cultural and Psychosocial factors**  individual / communal / community / gender / sex / culture / trauma / generation /age / social class / socio economic status/ social support / family support / resilient / resilience / protective / adaption |

Searches were conducted in 2022 and updated in September 2023. Screening was facilitated by Covidence software for reviews.

**Stage 3:** Select Studies; Inclusion and exclusion criteria

**Table 2**

Inclusion and Exclusion Criteria

| **Inclusion** | **Exclusion** |
| --- | --- |
| 1. Arab refugees, including displaced and other vulnerable populations, civilians | 1. non-Arab refugees, or military personnel |
| 1. adults 21 years of age and older | 1. children and youth under 21 |
| 1. participants have been exposed to violence assessed for and/or diagnosed with trauma-related distress and/or PTSD | 1. lack of violence exposure |
| 1. studies including any of the gender, class, generation (age), and cultural concept of self (GCG-CCS) variables in trauma conceptualization; or looked at local idioms of distress | 1. study has focused exclusively on psychiatric/counseling/medical treatments/interventions; or on psychometric properties and cross-cultural validation PTSD assessment instruments without exploring local idioms |
| 1. empirical qualitative and or quantitative research articles, knowledge synthesis such systematic reviews, meta-analyses, etc. | 1. book chapters, editorials, commentaries; unfiltered information (case studies, case reports, cohort reports) |
| 1. peer-reviewed papers published during 1990- 2023 | 1. published before 1990 |
| 1. published only in English and or Arabic languages; | 1. published in languages other than English or Arabic |
| 1. studies carried out in the 22 countries of the Arab Region; or reviews including studies carried out in the region | 1. studies carried out exclusively outside of the Arab Region |

*Interrater reliability*

Following the suggestion of (Tricco et al., 2016) we included an assessment of interrater reliability to verify the process validity of our review. Interrater reliability *(Cohen’s kappa)* of the screening phase by title and abstract: k value: [.86].
